# Applications and implementation of generative artificial intelligence in cardiovascular imaging with a focus on ethical and legal considerations: what cardiovascular imagers need to know!

**DOI:** 10.1093/bjrai/ubae008

**Published:** 2024-05-30

**Authors:** Ahmed Marey, Kevin Christopher Serdysnki, Benjamin D Killeen, Mathias Unberath, Muhammad Umair

**Affiliations:** Alexandria University Faculty of Medicine, Alexandria, 5372066, Egypt; Rowan-Virtua School of Osteopathic Medicine, Stratford, NJ, 08084, United States; Department of Computer Science, Laboratory for Computational Sensing and Robotics, Johs Hopkins University, Baltimore, MD, 21231, United States; Department of Computer Science, Laboratory for Computational Sensing and Robotics, Johs Hopkins University, Baltimore, MD, 21231, United States; Russell H. Morgan Department of Radiology and Radiological Sciences, The Johns Hopkins Hospital, Baltimore, MD, 21231, United States

**Keywords:** imaging AI, imaging AI policy, imaging AI implementation, AI ethics, automation, black-box problem, generative AI

## Abstract

Machine learning (ML) and deep learning (DL) have potential applications in medicine. This overview explores the applications of AI in cardiovascular imaging, focusing on echocardiography, cardiac MRI (CMR), coronary CT angiography (CCTA), and CT morphology and function. AI, particularly DL approaches like convolutional neural networks, enhances standardization in echocardiography. In CMR, undersampling techniques and DL-based reconstruction methods, such as variational neural networks, improve efficiency and accuracy. ML in CCTA aids in diagnosing coronary artery disease, assessing stenosis severity, and analyzing plaque characteristics. Automatic segmentation of cardiac structures and vessels using AI is discussed, along with its potential in congenital heart disease diagnosis and 3D printing applications. Overall, AI integration in cardiovascular imaging shows promise for enhancing diagnostic accuracy and efficiency across modalities. The growing use of Generative Adversarial Networks in cardiovascular imaging brings substantial advancements but raises ethical concerns. The “black box” problem in DL models poses challenges for interpretability crucial in clinical practice. Evaluation metrics like ROC curves, image quality, clinical relevance, diversity, and quantitative performance assess GAI models. Automation bias highlights the risk of unquestioned reliance on AI outputs, demanding careful implementation and ethical frameworks. Ethical considerations involve transparency, respect for persons, beneficence, and justice, necessitating standardized evaluation protocols. Health disparities emerge if AI training lacks diversity, impacting diagnostic accuracy. AI language models, like GPT-4, face hallucination issues, posing ethical and legal challenges in healthcare. Regulatory frameworks and ethical governance are crucial for fair and accountable AI. Ongoing research and development are vital to evolving AI ethics.

## Introduction

Artificial intelligence (AI) has become a prominent field in computer science, developing systems that mimic or surpass human intelligence.^1^^,^^2^ Machine learning (ML), a core AI technology, enables systems to learn from data and make predictions, including supervised, unsupervised, and reinforcement learning.^3^ Deep learning (DL), a subset of ML, utilizes multilayered neural networks to discern complex data patterns, mirroring the architecture of the human brain. Notable DL models include autoencoders, generative adversarial networks, convolutional neural networks (CNNs), recurrent neural networks, multilayered perceptions, neural radiance fields, and deep neural networks.^4^

Generative AI, a subset of ML, excels in producing data akin to the original dataset, facilitating applications such as image and speech synthesis. These models conduct unsupervised learning, extracting insights from data without explicit labels.^5^ This capability is valuable when labeled data is scarce or expensive to obtain. Generative AI models can fabricate synthetic data by understanding data distributions and generating new data that mirrors real-world statistics, distinguishing them from discriminative models that focus on classification and regression tasks.^5^ Generative AI encompasses various categories, each tailored to specific applications.^6^

This article explores generative artificial intelligence (GAI) applications and implementation in the setting of cardiovascular imaging. In CVS imaging, GAI holds promise for enhancing diagnostic accuracy, optimizing treatment planning, and predicting patient outcomes.^3^^,^^7^ Our objective is to provide an overview of ML- and DL-based GAI models in cardiovascular imaging, exploring their applications, recent advancements, challenges. Furthermore, we will address pertinent ethical and legal considerations surrounding the clinical implementation of GAI in cardiovascular practice. By elucidating these complexities, we aim to empower cardiovascular imagers to navigate the evolving AI landscape responsibly and ethically.

## AI applications in cardiovascular imaging

Generative AI (GAI) finds diverse applications across cardiac imaging modalities. In Cardiac MRI (CMR), GAI assists in image reconstruction by generating high-resolution images from undersampled data and automating segmentation tasks for precise volumetric and functional analysis. Additionally, GAI enhances CMR images through techniques like super-resolution, improving visualization of fine details.^8^ GAI also facilitates the quantification of metabolites in Cardiac MRI Spectroscopy, assessing myocardial health.^9^ In Cardiac Computed Tomography (CCT), GAI-based denoising methods enhance image quality by reducing noise and correcting artifacts, ensuring clearer visualization of cardiac structures.^10^ Echocardiography benefits from GAI by improving image quality through noise reduction and contrast enhancement, while also automating measurements of cardiac parameters for diagnosis and monitoring.^11^

### Echocardiography

Echocardiography, the most widely used imaging modality for cardiac diagnosis, faces challenges in standardization and operator-dependent variations.^12–15^ AI-empowered echocardiography aims to standardize interpretation, reduce operator-interpreter variations, and assist in cardiac disease identification.^11^^,^^14^^,^^15^ While various forms of AI have been used, ML methods required laborious manual annotation and exhibited low accuracy across equipment vendors.^12^^,^^13^ The most successful AI-based echocardiography approaches utilize DL with CNNs.^12^^,^^13^ DL systems accurately perform LV segmentation and calculate LVEF.^16–21^ Beyond segmentation, DL systems identify heart failure, cardiac amyloidosis, and pulmonary arterial hypertension, providing valuable insights for clinicians.^16–21^ However, generalizability remains a concern due to the relatively small datasets studied.^14^

### Cardiac MRI

Cardiac MRI is routinely used for clinical decision-making involving diagnosis, follow-up, and procedural planning.^8^^,^^22^ While CMR has significant potential to noninvasively visualize cardiac structure without radiation, long scan times and motion artifacts hinder efficacy, especially for 3D whole-heart data.^8^^,^^22^ Techniques like parallel imaging (PI) and compressed sensing (CS) aim to acquire fewer samples in the k-space, enabling faster acquisition but introducing aliasing artifacts.^22^^,^^23^ Advanced reconstruction methods, such as variational neural networks (VNNs), recover sparsely sampled k-space data, achieving high-quality reconstructions swiftly.^24^

Typically, CMR images are obtained over multiple breath-holds to minimize respiratory motion artifacts, which limits the data acquisition window to approximately 15-20 s in healthy patients.^22^^,^^24–26^ Ghodrati et al trained an adversarial autoencoder (AE) to reduce blurring and ghosting artifacts, enabling free-breathing scanning.^26^ DL-based reconstruction methods can significantly reduce scan times, minimizing patient discomfort, and potential motion artifacts.^27^ The high-quality reconstructions from undersampled data facilitate accurate diagnosis, procedural planning, and follow-up assessments.^28^ Overall, the utilization of undersampling techniques and advanced reconstruction algorithms holds promise for accelerating and enhancing CMR imaging.^22–26^

### Coronary CT angiography and CT morphology and function

The synergy of non-invasive CT imaging and ML is catalyzing a paradigm shift in the diagnosis of coronary artery disease (CAD). Coronary CT angiography (CCTA) serves as a pivotal tool in the non-invasive evaluation of CAD, with a negative predictive value of up to 99%. CCTA enables the assessment of stenosis severity and the quantitative analysis of atherosclerotic plaque characteristics.^29^^,^^30^ Integrating ML techniques, like Guner et al’s ensemble of artificial neural networks (ANNs), yields a model accuracy of 0.74, surpassing some non-specialist clinicians.^31^ Zreik et al incorporated recurrent CNNs into the analysis of multiplanar reformatted CCTA images, achieving accuracies of 0.77 and 0.8 in plaque and stenosis characterization, respectively.^32^

Fractional Flow Reserve CT (FFR-CT) evaluates coronary hemodynamics with strong concordance to invasive FFR but involves complex Computational Fluid Dynamics (CFD) calculations.^33–35^ Improvements in existing FFR-CT algorithms and/or better new models in the future can replace resource-intensive CFD computations, significantly reducing analysis time.^36–38^ For instance, Itu et al’s AI algorithm, trained on 12 000 coronary artery structures, achieved 83.2% accuracy in predicting FFR values and reduced analysis time from 196.4 to 2.4 s.^37^

Automatic scoring of coronary artery calcium (CAC) on cardiac CT scans is an independent predictor of cardiovascular events and mortality. Manual measurement of CAC is time-intensive and susceptible to inaccuracies. DL techniques automate this task, leading to substantial time savings and enhanced accuracy.^39–42^ ML models developed by van Assen et al and Wolterink et al provide consistent and robust quantification of calcification.^43^ Wolterink et al demonstrated that CAC quantification could be automatically derived from CCTA or non-enhanced cardiac CT scans, simplifying the clinical workflow and reducing radiation exposure.^40^ Functional imaging approaches in cardiac CT, such as myocardial infarction diagnosis, are gaining prominence with ongoing advancements in imaging and postprocessing technologies.^44–46^

### Structural CT of heart

Whole-heart segmentation in non-contrast CT (NCCT) is challenging due to poor tissue contrast, lack of clear reference standards, over-segmentation or omission, unbalanced foreground-background distribution, and variability in patient anatomy.^47–49^ Multi-atlas methods have been explored, but manual segmentation criteria can be ambiguous.^50–52^ Segmentation on CCTA is more successful, and methods to transfer reference segmentations to NCCT are being developed.^10^^,^^53^^,^^54^

Bruns et al used dual-energy CT to align CCTA and virtual non-contrast (VNC) images, training a CNN on segmented VNC images to achieve high accuracy in NCCT segmentation with a Dice Similarity Coefficient (DSC) score of 0.897 ± 0.034.^40^^,^^47^ Morris et al improved segmentation by coupling cardiac CT and CMR data, training a DNN for better accuracy than atlas-based methods.^55^

For native valve characterization and pre-operative surgical planning, Shirakawa et al used a CNN to generate 3D surgical heart models from cardiac CT images, reducing segmentation time significantly.^56^^,^^57^

### Congenital heart CT

Congenital Heart Disease (CHD), affecting 1 in 110 births in the US, presents complex variations in heart structure and artery connections.^58^ Xu et al developed a dataset of 110 3D CT images covering various CHD types and a baseline framework for CHD classification, achieving 82.0% accuracy.^59^

3D printing aids clinical decision-making and planning in CHD but requires detailed heart and vessel segmentation. Xu et al leveraged DL and graph algorithms to handle structural variations. Their method improved segmentation accuracy, with an 11.9% increase in the Dice Similarity Coefficient (DSC) score compared to existing methods.^60^ Validation with 3D-printed models confirmed the clinical applicability of their approach.^60^

## Ethical considerations

While growing utilization of GAI in cardiovascular imaging offers significant advancements, it raises important ethical considerations that need to be carefully addressed.^61^ Here, are several proposed critical ethical considerations associated with using GAI in cardiovascular imaging:

### Considerations around transparency and explainability 

DL models, such as CNNs, often operate input data in a way that no human interpreter, even the initial programmer, can easily explain.^61^^,^^62^ This is referred to as the “black box” problem of AI. The interactions and transformations happening within deep models are difficult to associate with explicit rules or logic.^63^ Parameters are optimized during the training process to minimize a loss function, which measures the discrepancy between predicted outputs and ground truth labels, for example. While this optimization leads to impressive performance, it makes it challenging to directly understand how each parameter influences the model's decision-making.^64^

In clinical practice, interpretability is crucial for physicians to trust and use GAI algorithms confidently. However, the “black box” problem limits human understanding and the reasoning behind a generated output.^4^^,^^8^^,^^65–67^ While complete understanding may prove impossible, evaluation metrics can and should be developed and readily available for clinicians to assess the performance of generative systems.

### Evaluation metrics

Evaluation metrics serve as quantitative measures to assess the performance and quality of generative systems. These metrics enable clinicians and researchers to evaluate and compare different GAI models, interpret their outputs, and gauge their suitability for clinical applications.

The most common evaluation metric used is area under the receiver operating characteristic (ROC) curve.^64^ The ROC curve represents the trade-off between the true positive rate (TPR) and the false positive rate (FPR) at different decision thresholds. Decision thresholds are the values that determine when a generative model predicts positive or negative values for classes based on the output of a binary classification test. By changing the decision threshold, we can adjust the trade-off between the TPR and the FPR of the model. The ROC curve plots the TPR against the FPR for different decision thresholds, showing how well the model can distinguish between desired and undesired outputs.^64^ A higher ROC score indicates better discriminative ability and higher quality in the generated samples.

These metrics offer a quantitative assessment of the generative model's ability to produce desired outputs and capture the underlying patterns of the training data. By evaluating ROC curves, clinicians can evaluate and compare generative models, leading to improvements in the quality and fidelity of the generated outputs.^64^

Some other important evaluation metrics for generative systems in cardiovascular imaging include:

1. Image Quality Metrics: These metrics assess the visual quality of the generated images. They include Peak signal-to-noise ratio (PSNR), Structural Similarity Index (SSIM), and Fréchet Inception Distance (FID).^68^
2. Clinical Relevance Metrics: These metrics assess whether generated outputs capture desired features, pathology characteristics, or clinical relevance based on expert annotations or guidelines.^68^^,^^69^
3. Diversity Metrics: Quantify the variability of generated outputs using metrics such as Inception Score (IS) and Mode Score (MS). IS evaluates the diversity based on the prediction confidence of a pre-trained classifier. MS quantifies the coverage of different modes or clusters in the generated data distribution.^70^
4. Quantitative Performance Metrics: These metrics are used to assess the accuracy in tasks like segmentation or detection. Examples are Dice Similarity Coefficient (DSC), Intersection over Union (IoU), and sensitivity/specificity.^71^
5. Human Perception Studies: In addition to objective metrics, subjective evaluation through human perception studies can provide valuable insights into the clinical usability and acceptability of generative systems. Expert reviewers or clinicians can provide qualitative feedback on the visual quality, clinical relevance, and overall usefulness of the generated outputs.^72^

As AI algorithms continue to evolve and new challenges emerge in cardiovascular imaging, there is a growing need for the continuous refinement and updating of evaluation metrics.^73^^,^^74^

AI algorithms, particularly in healthcare, are sensitive to changes in the environment and are liable to performance decay.^73^ Therefore, even after their successful integration into clinical practice, these algorithms should be continuously monitored and updated to ensure their long-term safety and effectiveness.^73^ This is particularly important in the field of cardiovascular imaging, where rapid advancements are being made.^75^

Ongoing research and development efforts are crucial in improving the evaluation and validation of generative models in clinical practice. For instance, a study developed a generative large language model, GatorTronGPT, for medical research and healthcare.^76^ The model was trained using a large amount of clinical and English text, and its utility for biomedical natural language processing and healthcare text generation was evaluated.^76^ The study found that synthetic NLP models trained using synthetic text generated by GatorTronGPT outperformed models trained using real-world clinical text.^76^

However, the adoption of a standardized and consistent evaluation methodology should be explored as an area of future work.^74^ This is because results from models that are similar in nature cannot be adequately compared if the criteria for the measurement and evaluation of these models are not standardized.^74^

Therefore, continuous refinement and updating of evaluation metrics, along with ongoing research and development efforts, are key to improving the evaluation and validation of AI algorithms, particularly in the field of cardiovascular imaging. These efforts will help ensure the long-term reliability and effectiveness of these algorithms in clinical practice.

As stated, perception studies and qualitative research can help provide nuance to objective evaluation metrics. Qualitative research can guide implementation by addressing what product dimensions affect social acceptability,^77^ and show imagers requirements to improve the usability of support systems.^78^ Moreover, qualitative studies can show cultural differences in perceptions of imaging AI implementation,^79–81^ and help develop patient-centred systems.^82^

By incorporating robust evaluation metrics, clinicians can gain a better understanding of the performance and limitations of GAI systems in cardiovascular imaging. These metrics can guide the adoption, interpretation, and integration of generative models into clinical workflows, enabling evidence-based decision-making and fostering trust in the AI algorithms used in patient care.

#### Automation and automation bias

Automation bias is the tendency for humans to unquestionably accept the output of an automated system without critical evaluation. Once an output is generated, clinicians may cease searching for confirmatory evidence and transfer decision-making responsibility onto the system.^83^ This bias arises from multiple factors, including a lack of understanding of AI limitations and the desire for efficiency and convenience, especially when overloaded with multiple tasks or nearing the end of a shift.^83^

#### Appropriate implementation

Because of the data involved, implementing AI systems requires adopting a digital health decision-making framework that considers ethical pillars, including respect for persons, beneficence, and justice.^84^ AI research has historically not required IRB approval, nor do technology companies have the medical expertise to evaluate the clinical appropriateness, utility, and implementation of AI in the clinic.^4^^,^^85^ For these reasons, quality control and assurance processes must be implemented to ensure the responsible and ethical use of patient data.^3^ Ethical roles that enable AI systems to be built are also required.

Ethical frameworks provide a structured approach to guide the development, deployment, and utilization of GAI systems in healthcare.^86^^,^^87^ Respect for persons acknowledges the autonomy and dignity of individuals, emphasizing informed consent and privacy protection through strict anonymization when using patient data. Beneficence underscores the obligation to maximize benefits and minimize harms, ensuring that GAI systems contribute positively to patient care. Justice focuses on equitable access, distribution, and fairness in the application of GAI technologies, avoiding biases and disparities.^86^^,^^88^

Historically, AI research, including GAI development, has often bypassed the need for Institutional Review Board (IRB) approval. However, the application of AI in clinical settings raises novel ethical and regulatory challenges. AI development often involves technology companies that may lack the medical domain knowledge required to evaluate the clinical appropriateness, utility, and potential impacts of the AI system.^4^^,^^84^ To address these challenges, robust quality control and assurance processes become imperative. These processes involve multidisciplinary collaborations. Rigorous validation of GAI systems should extend beyond technical performance metrics to encompass clinical relevance and safety.^89^

Transparency is key to building trust among clinicians, patients, and regulatory bodies. Developers of GAI systems should provide clear documentation of the model's architecture, training data, and performance metrics. Moreover, regular updates, refinements, and mechanisms to address emerging ethical and clinical challenges should be in place.

AI developers bear the responsibility of addressing ethical considerations throughout the GAI system's lifecycle. This includes collaborating with medical professionals to ensure that the AI system aligns with clinical needs, considering potential biases, and minimizing the potential for unintended harm. The process of integrating AI systems into clinical workflows must be gradual and collaborative, involving thorough validation and adaptation to ensure patient safety and optimal outcomes.^90^ This comprehensive approach ensures that GAI technologies become valuable tools for clinicians while prioritizing patient well-being and regulatory compliance.

#### Health disparities and diversity of training models

Vulnerable groups have a long-standing history of being underrepresented or entirely absent from existing datasets.^91^ If AI algorithms are trained on unbalanced datasets, the diagnostic and therapeutic utility of an AI system may be severely limited for those populations.^91^ Such algorithms may fail to properly diagnose entire groups, such as ethnic and gender minorities, immigrants, children, the elderly, and those with disabilities.^91^

In cardiac imaging, for example, Puyol-Antón et al performed the first analysis for sex and racial bias in cine CMR imaging segmentation using a large-scale database.^92^ After controlling for confounders, statistical analysis conducted showed that race was the main factor explaining why their DL-based cine CMR imaging segmentation model was less accurate for determining the biventricular volumetric and functional measurements in minority ethnic groups compared to the majority patient population.^92^ They concluded that although the UK Biobank imaging database they used was sex-balanced, the fact that it is not race-balanced limited the utility of their model for minority groups, especially for Chinese, mixed, and Black racial groups.^92^

#### Hallucination in GPT-4 and the potential ethical and legal ramifications

AI language models, like GPT-3.5 and GPT-4, exhibit a phenomenon known as hallucination or fabrication, producing outputs that may sound reasonable but are not factually accurate. These instances arise due to the limitations of training data and the model's inability to distinguish between correct and incorrect information.^93^ In one study evaluating the ability of GPT-3.5 to generate scientific references, it was observed that some references provided were erroneous or nonexistent, constituting partial or total AI hallucinations.^94^ While GPT-4 is an improvement over GPT-3.5, it is important to acknowledge that the challenge of hallucination remains a topic of discussion in the field and raises significant legal and ethical concerns.^95^^,^^96^

The phenomenon of hallucination undermines user trust and confidence in the generated outputs. This can impact the willingness of users to rely on AI systems and make informed decisions based on their outputs.^95^^,^^96^ Hallucination also poses ethical challenges. When AI systems generate false or misleading information, it can lead to misinterpretation or incorrect decision-making. In critical domains such as healthcare, relying on erroneous information can have severe consequences for patient care and safety.^97^

From a legal perspective, hallucination in AI systems can raise liability issues. If the generated information leads to harm or adverse outcomes, the responsibility and accountability for those consequences need to be determined. The legal frameworks surrounding AI systems are still evolving, but it is essential to consider the potential ramifications and allocate responsibility appropriately to ensure accountability and patient safety.^98^^,^^99^

Addressing the issue of hallucination in AI systems requires a multifaceted approach. Improving training inputs is crucial, including the use of diverse and contextually relevant datasets. Additionally, incorporating user feedback can help evaluate the AI-generated outputs, identify instances of hallucination, and provide corrective measures.^95^^,^^96^ Finally, having the model recheck its work has been shown to be beneficial too.^100^ Researchers and developers are actively working on improving AI models to minimize hallucination and enhance their reliability. Techniques such as prompt engineering,^101^ reinforcement learning from human feedback,^79^ and model fine-tuning are being explored to mitigate this issue.^102^

#### Healthcare data handling by the industry

In the realm of AI, particularly within healthcare, the preservation of data protection and privacy stands as a paramount concern. The increased utilization of algorithms for interpreting and making decisions based on healthcare data brings forth significant ethical implications, requiring a delicate balance between innovation and safeguarding individual rights and autonomy.^102^^,^^103^

### Regulation and accountability for fair and accountable AI

To cultivate fair and accountable AI practices within healthcare, a regulatory framework becomes essential. This includes methods for certifying AI systems, mechanisms for explaining their decisions, and processes for auditing their behaviour. Such regulations aim to strike a balance between innovation and patient safety, aligning AI advancements with ethical standards.^104^

### Ethical implications and governance of algorithm design

The ethical implications of AI in healthcare extend beyond just the technology itself. Algorithm design, development, and deployment require careful governance to ensure that the entire lifecycle of an AI system adheres to ethical principles. Scrutiny is essential to identify potential biases, discrimination, and unfairness that may inadvertently emerge from algorithmic decision-making. Ethical governance extends to the selection of training data, the calibration of algorithms, and ongoing monitoring for unintended consequences.^105^

### Mitigating discrimination while preserving privacy

A significant challenge in healthcare data handling lies in mitigating algorithmic discrimination without infringing upon individuals' privacy. A potential solution involves the involvement of trusted third parties. These entities can play a pivotal role in selectively storing and managing sensitive data while preserving privacy through techniques like differential privacy. This approach allows the extraction of valuable insights without compromising the privacy of individual patients.^106^

Given the intricate and changeable nature of the legal exposure that AI developers and maintainers in the private sector might face while handling large amounts of patient data, it will be essential to create well-crafted agreements outlining the rights and responsibilities of all parties involved, as well as establishing accountability for potential adverse outcomes.^107^

One potential method for AI system developers to address persistent privacy concerns is by utilizing generative data. Generative models have the capability to produce plausible yet fabricated patient data that is not linked to actual individuals.^108^ This approach could facilitate ML without the prolonged utilization of authentic patient data, even though initial use of such data might be necessary to develop the generative model.

### Ongoing need for research and development

Amid these discussions, it's important to acknowledge that the field of AI ethics is constantly evolving. The dynamic landscape of technology necessitates ongoing research and development to comprehensively address ethical challenges in healthcare data handling. This entails not only the advancement of algorithms themselves but also the continuous refinement of ethical frameworks, regulatory guidelines, and accountability mechanisms.^105^

Data protection and privacy in the context of AI, particularly within healthcare, demand a careful and holistic approach. Ensuring ethical considerations, robust regulation, the engagement of trusted third parties, and ongoing research is essential to cultivate fair, accountable, and patient-centric AI systems. By harmonizing technological advancements with ethical principles, the healthcare industry can harness the potential of AI while safeguarding individual rights, patient well-being, and the integrity of medical practice.

## Funding

The authors received no financial support for the research, authorship, and/or publication of this article.

## Conflicts of interest 

The authors declared no potential conflicts of interest with respect to the research, authorship, and/or publication of this article.
